# Editorial: Experimental Bias in Electrophysiological Studies

**DOI:** 10.1523/ENEURO.0432-17.2017

**Published:** 2017-12-20

**Authors:** Christophe Bernard

When writing about the experimental method, Claude Bernard stated:

We make an observation or an experiment. But once observations and experiments have been performed, we reason about them. This is when any type of explanation can be produced according to everyone’s way of thinking.

The observation is what it is, a fact, but its interpretation depends on the conceptual framework we are using. This framework is based on what we think we know at time *t*. Since our understanding of phenomena is constantly evolving, it is not surprising to find numerous examples in science when the most appropriate data interpretation had to wait years/decades following the initial observations. We all accept this, because this is how Science progresses. But there are more insidious traps in data interpretation, e.g., confounding factors that we are not aware of, or interpretations that we take for granted. Alerting on these experimental biases and common mistakes in data interpretation is also a goal of *eNeuro*, as we are here to serve the scientific community and believe highlighting these issues is an important step to avoiding these pitfalls. This editorial will hopefully spawn an ongoing series where we, scientists, will highlight these issues in the various Neuroscience subfields to the benefit of all.

I will start with a rather common problem in electrophysiological research in interpreting local field potentials. Many studies use the recording of local field potentials to investigate brain function and dysfunction. However, interpreting a local field potential, i.e., what is actually measured, is not as straightforward as it seems. On this topic, I recommend reading the excellent review in *Nature* by Buzsáki et al. In short, brain cells that allow ion flow through their membrane can generate an electrical field, usually in the form of a sink/source dipole. The dipole is the result of charged particles passing through the membrane at a specific location and other charges crossing at a different membrane location. In cortical structures, pyramidal cells are spatially organized, their dendrites being more or less aligned. As a result, sources and sinks tend to be spatially aligned, allowing local fields to add up and be detected by a recording electrode.

However, in regions like the striatum, there is no geometrical arrangement allowing the summation of fields. If a dendrite of a medium spiny neuron is active, it will generate a small dipole, locally, with a given orientation. Since dendrites are not spatially aligned in the striatum, dipoles may have any type of orientation, resulting in a global net null electrical field. A way to increase confidence that the recorded field is local is to use a bipolar electrode and to use one lead as the reference (although it can also lead to false positives).

It is possible to record a local field potential in nonlayered structures, like the striatum. Many studies correlate such fields to behavioral outputs, or their alterations to some pathologic conditions. Given the lack of geometrical organization, it is difficult to imagine a condition allowing the genesis of a large field. An alternative explanation is volume conduction. When a large field is generated, it can propagate and be detected far from its source. This is the reason why we can record EEG on the skull. Thus, volume conduction may explain field potentials recorded in nonlayered structures. Two papers recently published, one in *eNeuro* and the other in *JNeurosci*, address this issue in the striatum. Lalla et al. show that θ oscillations recorded in the striatum are most likely volume conducted from the hippocampus. Carmichael et al. show that striatal γ oscillations are volume conducted from the piriform cortex.

This does not mean that the interpretations resulting from field potential recordings performed in nonlayered structures are to be discarded. Reasons not to discard striatal local field potential information altogether include the observation that spike timing in many striatal neurons is related to local field potential phase, and ensemble spiking activity can be used to predict frequency. However, measures (e.g., as described in these two papers) must be taken to determine whether the fields are indeed local. Carmichael et al. showed that nostril occlusion abolished γ oscillations, demonstrating their piriform cortex origin. Assessing the source of the field is very important, because a wrong interpretation may lead researchers astray. For example, many studies report changes in power of θ/γ local field potential oscillations in specific physiologic and pathologic conditions in the striatum. Investigating the mechanisms underlying these modifications require looking in the structures generating these fields (i.e., outside the striatum) rather than where they are recorded.

Unfortunately, there is no ideal solution to address the problem of the interpretation of field potentials. A bipolar montage, or more generally, current source density analysis, may help to determine whether the signal is local. But it must be stressed here that interpreting a field in the brain corresponds to an inverse problem with an infinite number of solutions, even in layered regions. Common sense helps us interpret the field that is measured in layered structures, but it remains common sense, i.e., not a sure thing. Complementary measurements can be used, such as spikes. Spikes may be a more reliable way to assess information flow between the striatum and other regions. Indeed, the difficulty in interpreting local field potentials recorded in nonlayered structures should not preclude the investigation of the coding potential of oscillatory patterns of activity. This seemingly paradoxical statement is illustrated by Lalla et al. and Carmichael et al., as both studies reported oscillatory spiking activity of single neurons at θ and γ frequencies in conjunction with volume-conducted local field potentials.

In conclusion, caution always needs to be exerted and a technique never taken for granted. For example, two other papers recently published in *eNeuro* by Steinmetz et al. and Hasegawa et al. alert us to the difficulty to interpret results when GCamp6 mice and luciferase shRNA are used.

If you feel like you need to communicate to the scientific community on the difficulty interpreting data (of any type), please contact me at eneuroeditor@sfn.org.

Cheers,

